# The effectiveness of relugolix compared with leuprorelin for preoperative therapy before laparoscopic myomectomy in premenopausal women, diagnosed with uterine fibroids: protocol for a randomized controlled study (MyLacR study)

**DOI:** 10.1186/s13063-024-08170-1

**Published:** 2024-05-24

**Authors:** Mari Kitade, Jun Kumakiri, Hiroyuki Kobori, Keisuke Murakami

**Affiliations:** 1 Juntendo University Faculty of Medicine, 2-1-1 Hongo, Bunkyo-Ku, Tokyo, 113-8431 Japan; 2 Department of Obstetrics and Gynecology, Tokyo Women’s Medical University, 8-1 Kawada-Cho, Shinjuku-Ku, Tokyo, 162-8666 Japan; 3 Medical Topia Soka Hospital, 1-11-18 Yatsuka, Soka-City, Saitama, 340-0028 Japan

**Keywords:** Uterine fibroids, Leiomyomas, Preoperative therapy, Intraoperative blood loss, Laparoscopic myomectomy, GnRH antagonist, Relugolix, GnRH agonist, Leuprorelin

## Abstract

**Background:**

The oral gonadotropin-releasing hormone antagonist relugolix, which temporarily stops menstruation, is used to treat heavy menstrual bleeding, pelvic pressure, and low back pain in women with uterine fibroids. Treatment can also help women recover from low hemoglobin levels and possibly shrink the fibroids. However, evidence of preoperative use of relugolix before laparoscopic myomectomy is limited. Nevertheless, the treatment could reduce interoperative blood loss, decrease the risk of developing postoperative anemia, and shorten the operative time. Thus, we aim to test whether 12-week preoperative treatment with relugolix (40 mg orally, once daily) is similar to or not worse than leuprorelin (one injection every 4 weeks) to reduce intraoperative blood loss.

**Methods:**

Efficacy and safety of preoperative administration of drugs will be studied in a multi-center, randomized, open-label, parallel-group, noninferiority trial enrolling premenopausal women ≥ 20 years of age, diagnosed with uterine fibroids and scheduled for laparoscopic myomectomy. Participants (*n* = 80) will be recruited in the clinical setting of participating institutions. The minimization method (predefined factors: presence or absence of fibroids ≥ 9 cm and the International Federation of Gynecology and Obstetrics [FIGO] type 1–5 fibroids) with randomization is used in a 1:1 allocation. Relugolix is a 40-mg oral tablet taken once a day before a meal, for 12 weeks, up to the day before surgery. Leuprorelin is a 1.88 mg, or 3.75 mg subcutaneous injection, given in three 4-week intervals during patient visits before the surgery. For the primary outcome measure of intraoperative bleeding, the blood flow is collected from the body cavity, surgical sponges, and collection bag and measured in milliliters. Secondary outcome measures are hemoglobin levels, myoma size, other surgical outcomes, and quality-of-life questionnaire responses (Kupperman Konenki Shogai Index and Uterine Fibroid Symptoms—Quality of Life).

**Discussion:**

Real-world evidence will be collected in a clinical setting to use pre-treatment with an oral gonadotropin-releasing hormone antagonist to reduce intraoperative bleeding in women who undergo laparoscopic myomectomy.

**Trial registration:**

jRCTs031210564 was registered on 19 January 2022 in the Japan Registry of Clinical Trials (https://jrct.niph.go.jp).

## Administrative information

Note: the numbers in curly brackets in this protocol refer to SPIRIT checklist item numbers. The order of the items has been modified to group similar items (see http://www.equator-network.org/reporting-guidelines/spirit-2013-statement-defining-standard-protocol-items-for-clinical-trials/).

**Table:** 

Title {1}	The effectiveness of relugolix compared with leuprorelin for preoperative therapy before laparoscopic myomectomy in premenopausal women, diagnosed with uterine fibroids: protocol for a randomized controlled study (MyLacR study)
Trial registration {2a and 2b}.	Trial identifier: jRCTs031210564 in the Japan Registry of Clinical Trials (jRCT) at https://jrct.niph.go.jp/en-latest-detail/jRCTs031210564
Protocol version {3}	Ver 5.0
Funding {4}	Relugolix and leuprolide acetate will be manufactured by ASKA Pharmaceutical Co., Ltd., Tokyo, Japan, and provide funding for the design, publication, meetings, and central organizational costs. ASKA Pharmaceutical Co., Ltd., Tokyo, Japan, is funding the running costs for the MyLacR study trial and recruitment of up to 80 women, as specified in the joint research contract with Juntendo University School of Medicine, Tokyo, Japan. Study management, analysis and reporting of the study are independent of the manufacturer of relugolix and leuprolide acetate.
Author details {5a}	Mari Kitade^1^, Jun Kumakiri^2^, Hiroyuki Kobori^3^ and Keisuke Murakami^1^ ^1^Juntendo University Faculty of Medicine, 2–1-1 Hongo, Bunkyo-ku, Tokyo 113–8431. Email address: kitade@juntendo.ac.jp ^2^Department of Obstetrics and Gynecology, Tokyo Women’s Medical University, 8–1 Kawada-cho, Shinjuku-ku, Tokyo 162–8666. Email address: kumakiri.jun@twmu.ac.jp. ^3^Medical Topia Soka Hospital, 1–11-18 Yatsuka, Soka-city, Saitama 340–0028. Email address: kobori@mtopia.jp. Corresponding author: Mari Kitade
Name and contact information for the trial sponsor {5b}	ASKA Pharmaceutical Co., Ltd., Medical Affairs Department 5–1 Shibaura 2-chome, Minato-ku, Tokyo 108–8532, Japan
Role of sponsor {5c}	ASKA Pharmaceutical Co., Ltd., Tokyo, Japan, provides funding for the design, publication, meetings, and central organizational costs but does not have ultimate authority over study management and data analysis, nor does the sponsor have control over the contents of the report or decision to submit the report for publication.

## Introduction

### Background and rationale {6a}

Uterine fibroids are common benign tumors in the uterine smooth muscle of reproductive-age women [1]. Patients may be asymptomatic, though some have heavy menstrual bleeding, prolonged menses, dysmenorrhea, pelvic pressure symptom, and anemia [1]. The severity of symptoms depends on the location, size, and number of fibroids. Also, uterine fibroids are sometimes responsible for infertility and early miscarriage [2, 3]. These significantly impact patients’ daily activities and can impair quality of life (QOL) [4, 5].

Uterine fibroids can be treated surgically or with pharmacotherapeutics [1]. Treatment choice depends on the patient’s age, symptoms, and the tumor’s number, size, and location. In addition, the patient’s desire to preserve fertility is vital in determining the treatment plan. Surgical treatments include total hysterectomy*,* myomectomy, endometrial ablation, uterine artery embolization, transcervical resection, and focused ultrasound surgery. There are three approaches to total hysterectomy and myomectomy: open, vaginal, and laparoscopic. Decades ago, uterine fibroids were considered a disorder in women past childbearing age; thus, a hysterectomy was standard care [6]. However, with the change in women’s lifestyles, such as late marriage and childbirth, women with fibroids may still want to have a child. Thus, myomectomy [1] is considered the preferred surgical procedure. Furthermore, compared to open myomectomy, the laparoscope procedure is preferred because patients usually do not develop a fever, have milder postoperative pain, and have a shorter hospital stay [7]. Thus, minimally invasive laparoscopic myomectomy (LM) is becoming the norm.

In women scheduled to undergo LM, a gonadotropin-releasing hormone (GnRH) agonist or GnRH antagonist may be administered to improve operative outcomes. These agents, as they reduce sex hormone levels and induce amenorrhea, help restore hemoglobin levels, shrink myomas, and improve myoma-related symptoms.

The 2017 Cochran Collaboration Systematic Review of therapy before surgery for uterine fibroids reported that GnRH agonist therapy compared to placebo, improved intraoperative and postoperative outcomes of LM [8]. These were a shorter operative time, a decrease in intraoperative blood loss that reduces the risk of developing postoperative anemia, and a decrease in uterine and fibroid volume. In addition, another meta-analysis of GnRH agonists versus placebo before LM reported a decrease in intraoperative blood loss and an increase in postoperative hemoglobin levels [9].

However, GnRH agonists such as leuprorelin induce an initial transient increase in GnRH levels (flare-up) with a subsequent reduction in sex hormones triggering GnRH receptor downregulation and desensitization. On the other hand, the competitive GnRH receptor antagonist relugolix does not trigger a flare-up, and its effects are immediate.

Relugolix is a small-molecule oral GnRH antagonist used to treat uterine fibroids and endometriosis in Japan. In a Japanese phase 3 study comparing relugolix and leuprorelin treatments in patients with uterine fibroids and heavy menstrual bleeding [10], the noninferiority of relugolix to leuprorelin in reducing the bleeding was confirmed. In addition, both groups had similar efficacies in reducing the size of uterine fibroids. Furthermore, the severity and incidence of adverse events (AEs) were similar in the relugolix and leuprorelin groups.

Japan Society of Gynecologic and Obstetric Endoscopy and Minimally Invasive Therapy (JSGOE) Guidelines for Endoscopic Surgery in Obstetrics and Gynecology [6] reports that the oral GnRH antagonist relugolix available in 2019 can now be prescribed to treat patients with uterine fibroids. Unlike GnRH agonists, this formulation does not cause a flare-up and can improve uterine fibroid symptoms and potentially reduce fibroid volume. However, preoperative use of relugolix in patients undergoing LM has not been tested in a randomized controlled trial (RCT).

### Objectives {7}

Thus, with the overall aim of improving operative outcomes of LM, the study’s hypothesis is to test the 12-week preoperative noninferiority of relugolix (40 mg orally, once daily) versus leuprorelin (1.88 mg, or 3.75 mg, three injections, once every 4 weeks) to reduce intraoperative blood loss.

Secondary objectives are to (1) assess other surgical outcomes besides intraoperative bleeding, (2) measure changes in fibroid size and uterine volume, (3) measure changes in hemoglobin levels, and (4) observe changes in menopausal-like symptoms, disease-specific QOL, and the safety of treatments.

### Trial design {8}

The design is a multi-center, randomized, parallel-group, noninferiority trial, which will be conducted as a “specified clinical trial” according to the Clinical Trials Act in Japan. Randomization will be performed using the minimization method with a 1:1 allocation of participants to receive relugolix or leuprorelin.

## Methods: participants, interventions, and outcomes

### Study setting {9}

Data will be collected at the department of gynecology and obstetrics at three hospitals in Japan: Juntendo University Hospital, Tokyo Woman’s Medical University Hospital, and Medical Topia Soka Hospital. The investigators (gynecological surgeons) will recruit potential candidates from patients scheduled to undergo LM.

### Eligibility criteria {10}

Patients must provide written, informed consent and meet all criteria before randomization.

Inclusion criteria

1. Age ≥ 20 years
2. Premenopausal women
3. Diagnosis of uterine fibroids by magnetic resonance imaging (MRI)
4.  ≤ 5 uterine fibroids, ≥ 2 cm at the largest dimension (MRI findings during screening)
5. The largest uterine fibroid diameter ≥ 4 cm and ≤ 12 cm (MRI findings during screening)
6. Scheduled for LM
7. Can give written, informed consent for participation in the study

Exclusion criteria

1. History of pelvic surgery (excluding cesarean section)
2. Complicating of ovarian cysts or endometriosis (diagnostic imaging)
3. Complicating of uterine adenomyosis
4. Sex hormones used within four weeks before the signing of the informed consent form
5. GnRH analogues used within 19 weeks (sustained-release formulation) or 15 weeks before the signing of the informed consent form
6. Contraindicated for laparoscopic surgery
7. Presence of malignant tumors
8. Coagulation abnormalities
9. Anticoagulant use
10. Suspected or confirmed pregnancy, or breastfeeding
11. Undiagnosed abnormal genital bleeding
12. Hypersensitivity to any component of relugolix tablets or leuprorelin for injection, LH-RH or LH-RH derivatives

### Surgeon criteria for performing laparoscopic myomectomies

The surgeon performing laparoscopic procedures in this study must be accredited by the JSGOE and have conducted more than 100 laparoscopic myomectomies.

### Who will take informed consent? {26a}

The investigators of participating institutions fully informed potential participants about the study before study initiation. After giving potential participants sufficient time to consider whether or not to participate, investigators obtained their written informed consent.

### Additional consent provisions for collection and use of participant data and biological specimens {26b}

We will request consent for review of participants’ medical records, collection of myoma samples, collection of blood samples to check hemoglobin pre- and post-preoperative drug treatment and blood chemistry, and use of the samples in future studies. In that case, the authors will request permission for this secondary use of data from the Certified Review Board. If a participant does not wish the data to be used this way, the data will not be placed on participating institutions’ websites or other websites. This use of personal data is protected.

## Interventions

### Explanation for the choice of comparators {6b}

Leuprorelin is indicated for the treatment of uterine fibroids in Japan and has been often used for preoperative therapy before LM.

### Intervention description {11a}

Relugolix group participants will take one oral relugolix 40 mg tablet daily before one meal for 12 weeks.

Leuprorelin group participants will receive a subcutaneous (SC) leuprorelin injection of 1.88 mg or 3.75 mg (according to body weight and degree of uterine enlargement) at the medical institution once every four weeks for 12 weeks as specified in Japan’s package insert [11].

The dosing will start within days 1 to 5 of the menstrual cycle in both groups. Patients in the relugolix group will receive tablets at 4-week intervals during visits 2, 3, and 4. They will take the medication at home according to the schedule and record any missed dose in the patient diary. Patients in the leuprorelin group visit the medical institution to receive the SC injection once every 4 weeks.

### Criteria for discontinuing or modifying allocated interventions {11b}

An investigator discontinues the allocated intervention for a trial participant for any of the following:

1. The trial participant withdraws consent
2. Inclusion criteria are not met or only met after enrollment
3. The appearance of an AE or disease justifying the discontinuation, as determined by the investigator
4. Exacerbation of primary disease
5. The trial participant has difficulty continuing the study due to logistical matters
6. Pregnancy
7. The investigator recognizes the need to discontinue the allocated intervention for other reasons

## Strategies to improve adherence to interventions {11c}

### Adherence assessments

Participants in the relugolix group will check a box in the patient’s diaries when a dose is missed. At each visit, the investigator checks the patient’s diary during the interview and enters the information on the case report form (CRF). Leuprorelin is administered during the patient visit, and participants who refuse to receive the injection will be noted on the CRF.

### Relevant concomitant care permitted or prohibited during the trial {11d}

Permitted concomitant medications

1. Drugs for symptomatic treatment of symptoms associated with uterine fibroids (e.g., iron, tranexamic acid, Chinese herbal medicine, tranquilizers)
2. Therapeutic agents for diseases other than uterine fibroids

All medications used will be recorded on the CRF. However, procedural medications such as surgery-related pre-anesthetic medications will not be recorded.

Prohibited concomitant medications

1. GnRH agonists and antagonists (excluding test and reference drugs)
2. Anticoagulants
3. Sex hormones
4. Oral contraceptives and intrauterine devices

### Provisions for post-trial care {30}

There is no provision for post-trial care. The outcome will be determined at the end of the study, and no further follow-up will be required. However, since participants are routine clinical patients at medical institutions, they will continue to receive treatment when required. The drugs are approved for use during routine clinical practice and are covered by National Healthcare Insurance. Patients who might experience AEs from trial participation are protected through the insurance of participating hospitals and National Healthcare Insurance.

### Outcomes {12}

#### Primary outcome measure

Intraoperative blood loss was selected as the primary outcome measure because postoperative anemia is a complication of LM. Also, pre-LM treatment with a GnRH agonist compared to no pre-treatment reduced the bleeding, as described in a 2017 meta-analysis [8]. Thus, 12-week pre-LM treatment with relugolix (test drug) or leuprorelin (active control) that results in reduced intraoperative blood loss in both treatment arms is classed as treatment success, defined further in the “Sample size {14}” section.

#### Secondary outcome measures

1. Surgical outcomes (operative time, extracted myoma weight and number, required blood transfusion or not, switch to open surgery or not, postoperative drainage volume, length of postoperative hospital stays)
2. Percent change from baseline in uterine fibroid size at the end of preoperative drug treatment (week 12)
3. Percent change from baseline in uterine volume at the end of preoperative drug treatment (week 12) and post-surgical week 12
4. Change from the predose value in hemoglobin at the end of preoperative drug treatment (week 12) and post-surgical day 1, week 4, and week 12
5. Change from the predose value in menopausal-like symptoms: Kupperman Konenki Shogai Index (KKSI) at week 4, week 8, and week 12 of preoperative drug treatment and post-surgical week 4 and week 12
6. Change from the predose value in disease-specific QOL: Uterine Fibroid Symptom Health-Related Quality of Life (UFS-QOL) at the end of preoperative drug treatment (week 12) and post-surgical week 12

#### Safety outcome measures

1. AEs; adverse drug reactions; intraoperative complications; postoperative complications
2. Blood pressure
3. Endocrinology (estradiol, progesterone, luteinizing hormone, follicle-stimulating hormone)
4. Blood chemistry (aspartate aminotransferase, alanine aminotransferase, triglycerides, total cholesterol, high-density lipoprotein cholesterol, low-density lipoprotein cholesterol)
5. Bone mineral density
6. Bone turnover markers (tartrate-resistant acid phosphatase-5b, bone-specific alkaline phosphatases)
7. Menstrual status (start and end dates)
8. Endometrial thickness
9. Histopathological examination of the extracted myoma, including examination for malignancy or cellular degeneration

### Participant timeline {13}

**Fig. 1:**
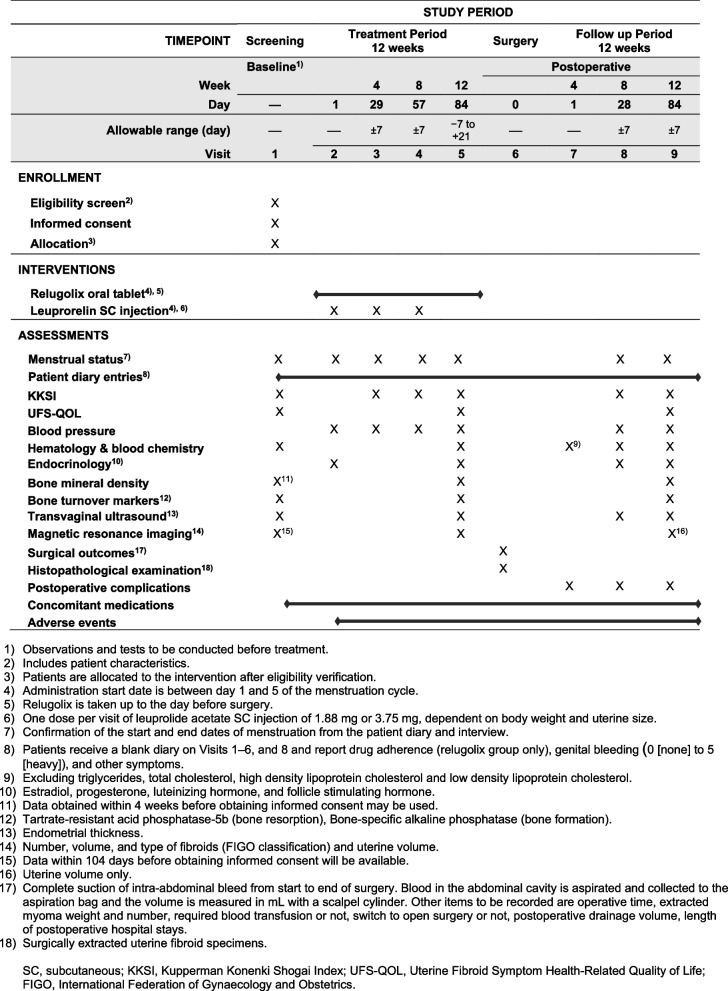
Schedule of eligibility screening, enrollment, group allocation, visits, interventions, and assessments for presurgical drug treatments and surgical intervention

The schedule of eligibility screening, enrollment, group allocation, visits, interventions, and assessments for presurgical drug treatments and surgical intervention is shown in Fig. 1.

### Sample size {14}

The research hypothesis for this study is that when comparing the amount of intraoperative blood loss during LM after receiving either relugolix or leuprorelin for 12 weeks, the intraoperative blood loss for the relugolix group is no more than 50 mL greater than that for the leuprorelin group. Treatment success is defined according to the single-center pilot study that compared pre-treatment with leuprorelin versus no pre-treatment for intraoperative blood loss during LM. In the study, blood loss was 90.0 ± 67.4 mL [mean ± standard deviation (SD)] for pre-treatment with leuprorelin (*n* = 51) versus 148.2 ± 122 mL for no pre-treatment (*n* = 52) (not published). An increase in intraoperative blood loss not only delays postoperative recovery but also leads to a reduction in surgical quality. Based on our clinical experience, we considered a difference of 50 ml or less as acceptable and, therefore, used 50 mL as the non-inferiority margin. With a noninferiority margin of 50 mL, a mean difference of 0 mL, SD of 70 mL, a one-sided significance level of 2.5%, and power of 80%, the required number of subjects for analysis was calculated to be 64 (32/group). A dropout rate of 20% was assumed; thus, the number of participants was set at 80 (40 in each group).

### Recruitment {15}

Investigators of participating hospitals identified potential trial participants from routine medical practice. All outpatients were asked if they wanted to participate when they were told that they were getting an operation. We recruited 1 or 2 subjects/center/month for 23 months. During the recruitment, no leaflet, poster, or any additional measure was used.

## Assignment of interventions: allocation

### Sequence generation {16a}

After the patients meet the study criteria and consent to participate, the investigator conducts screening tests and enters the patients’ data into the electronic data capture (EDC) system (SmartStage).

The EDC system determines eligibility based on input content and then randomizes the patients with the minimization method and 1:1 allocation. Adjustment factors are surgeon, the presence or absence of fibroids ≥ 9 cm in the largest dimension, and the presence or absence of the International Federation of Gynaecology and Obstetrics (FIGO) classification of fibroid types 1 to 5.

### Concealment mechanism {16b}

Allocation concealment will be ensured as the EDC system will not release the randomization code until the patient has been recruited into the trial.

### Implementation {16c}

Investigators will input patients’ registration data in the EDC system and enroll patients. The EDC system generates the allocation sequence and provides a registration number and treatment-arm allocation for each eligible participant.

## Assignment of interventions: blinding

### Who will be blinded {17a}

Blinding will not be performed for this study because the administration methods are an oral tablet versus an SC injection, and placebo is not administered. Furthermore, surgeons will not be blinded because they may examine patients during the preoperative visits. Thus, it is an open-label study. In addition, the person in charge of statistical analyses will access to the database after data cleaning and database lock and perform analyses according to a predetermined statistical analysis plan.

### Procedure for unblinding if needed {17b}

The study is open-label. Therefore, unblinding is not required.

## Data collection and management

### Plans for assessment and collection of outcomes {18a}

Investigators will collect data from study participants during nine visits: visit 1 for baseline data collection, visits 2 to 5 (12 weeks of treatment), visits 6 and 7 during hospitalization for surgery, and visits 8 and 9 (12 weeks of follow-up), for a study that will be completed in about 24 weeks. The planned schedule for data collection is shown in the “Participant timeline {13}” section.

Baseline, outcomes, and other trial collection and assessment data are directly recorded on the CRF. The investigator enters all data into the EDC system. The reliability and validity of the CRF are checked in the EDC system by EPS Corporation, the study’s contract research organization (CRO).

### Plans to promote participant retention and complete follow-up {18b}

Since the study is conducted as a part of standard care, investigators shall inform participants that research funding will cover payments of any uninsured portions of treatments.

At each visit, participants will receive a cash card of 1000 yen to cover travel or miscellaneous expenses, which is noted in the logbook. No other compensation is planned.

### Data management {19}

Registration and data collection in the CRF use an EDC system. The investigator will enter the required, anonymized trial subject data into the EDC. Research collaborators may be involved in EDC entry if only source data is identified as being recorded by them. The EDC users have individual IDs and passwords for authentication and access and encrypted data communications.

In this study, data management is performed by EPS Corporation. The sequence of procedures from EDC data entry validation and details of quality control are specified in the Data Management Plan.

### Confidentiality {27}

The investigator shall archive the documents related to the conduct of the research at each participating medical institution for 5 years. Afterwards, the materials are disposed of and identified as “processed.” Thus, the data cannot be traced back to trial participants.

### Plans for collection, laboratory evaluation, and storage of biological specimens for genetic or molecular analysis in this trial/future use {33}

Biological specimens will not be collected, evaluated, and stored for genetic or molecular analysis in this trial.

## Statistical methods

### Statistical methods for primary and secondary outcomes {20a}

Summary statistics (number of patients, mean, SD, minimum, and maximum quartiles) and two-sided 95% confidence interval (CI) will be calculated for the primary outcome of interoperative blood loss for the full analysis set (FAS) or, if appropriate, the per-protocol set (PPS) for each treatment group. The FAS will include participants who received at least one dose of treatment after randomization and underwent LM with assessment of the primary outcome. The PPS will be the target population from the FAS, excluding trial participants who deviate from or violate inclusion criteria, take less than 80% of medications, or take prohibited concomitant medications.

Noninferiority of the test-drug group (relugolix) to the active-control group (leuprorelin) is demonstrated when the upper limit of the two-sided 95% CI for the difference in intraoperative blood loss between the groups is ≤ 50 mL. Student’s *t*-test will be used to conduct the noninferiority test with a margin of 50 mL and a one-sided significance level of 2.5%.

For secondary outcomes, categorical data will be used to calculate each category’s numbers and percentage for each treatment group’s analysis population. The Wilcoxon rank sum test will compare ordinal data, and items observed in the nominal category are compared between the two groups by the chi-square (*χ*^2^) test. Fisher’s exact test will be used for binary values. Continuous data will be summarized for each treatment group (number of patients, mean, SD, minimum, quartile, and maximum). In addition, summary statistics (sample size, mean, SD, minimum, quartile, and maximum) of the change from baseline (screening phase) for each visit will be calculated, and the two groups will be compared by Student’s *t*-test or Wilcoxon rank sum test.

### Interim analyses {21b}

Interim analyses will not be conducted.

### Methods for additional analyses (e.g., subgroup analyses) {20b}

Subgroup analyses will not be conducted. However, when baseline factors differ between groups and might affect the primary outcome, an analysis of covariance (ANCOVA) with the factor as a covariate will be considered.

### Methods in analysis to handle protocol non-adherence and any statistical methods to handle missing data {20c}

The Case Review Committee will discuss procedures for handling missing data. The data will be classified as missing, and no imputation method will be used.

### Plans to give access to the full protocol, participant level-data and statistical code {31c}

The complete study protocol in Japanese is available upon reasonable request to the corresponding author.

## Oversight and monitoring

### Composition of the coordinating center and trial steering committee {5d}

The chief investigator has contracted with the clinical research organization, EPS Corporation, to be the coordination center of the study. The company provides overall management, monitoring, auditing (if performed), data management, statistical analysis, and medical writing services. In addition, the CRO will periodically submit reports on their work progress and results to the chief investigator. Meetings between the chief investigator, investigators at participating institutions, and CRO representatives will be held when required.

### Composition of the data monitoring committee, its role and reporting structure {21a}

A designated monitor by the principal investigator will visit each participating hospital and verify the source document of the first registered participant and report the monitoring results to the principal investigator. Monitoring of the second and subsequent participants will be conducted as needed. Monitoring will be carried out by EPS Corporation, independently of the funder, ASKA Pharmaceutical Co., Ltd.

### Adverse event reporting and harms {22}

Any expected or unexpected AEs related to the conduct of research include abnormal laboratory values or symptoms, in addition to disease, disability, infection, or death. Expected AEs are those listed in the package inserts of test and control drugs. When possible, the relationship with the AE and test drug or procedure will be specified as one of the following: not related or due to surgery, test drug, control drug, concomitant medications, other, or unknown. The AEs categorized as unknown are those in which the incidence, frequency, and conditions of the AE are not described in package inserts of test and control drugs, research protocol, and informed consent form.

The investigator shall state in the CRF the name of the disease, date of onset, severity level, treatment details, and outcome. When an investigator becomes aware of a serious illness or other AE in the conduct of the clinical study, the investigator shall promptly report this to the supervisor (chief medical officer) of the participating medical institution and the chief investigator, who shall report this to the Certified Review Board, provide information to other investigators, and promptly report the content of such information to the administrators of the participating medical organizations. The schedule for reporting is described in the complete protocol.

### Frequency and plans for auditing trial conduct {23}

Auditing is not planned for this study, though if required, auditing will be carried out by EPS Corporation, the CRO of the study. EPS will also manage the study by reviewing monthly progress and setting up meetings as needed.

### Plans for communicating important protocol amendments to relevant parties (e.g., trial participants, ethical committees) {25}

Study protocol revisions shall be made according to procedures stipulated by the Certified Review Board. The chief investigator will add a listing of revisions with reasons on the protocol’s cover, update the protocol’s date and version, and report changes in the jRCT, owned and managed by the Ministry of Health, Labour and Welfare of Japan at https://jrct.niph.go.jp/.

Information about the study is registered in the jRCT and updated as the research progresses. EPS Corporation representatives will prepare a summary of the primary outcome report within 1 year of the date of completion of the collection period for the primary outcome data and a summary of the clinical study report and clinical study report within 1one year of the date of completion of the collection period for all data and release the report to the jRCT within 1 month of the date of review by the Certified Review Board.

### Dissemination plans {31a}

The results obtained in this study will be presented at scientific meetings, published as papers in professional journals, and deposited in a public database. In any case, published results should only be statistically processed, and no personal information of the trial participants will be published.

## Discussion

This MyLacR study is expected to provide high-level evidence for preoperative relugolix to improve LM surgical outcomes, mainly to decrease intraoperative blood loss and the risk of postoperative anemia. Patient recruitment has been ended on 31 December 2023, and we did not have any practical or operational issues while carrying out the study during the recruitment.

Intraoperative blood loss is the primary outcome measure because it affects LM recovery. Menopause-like symptoms (measured with the KKSI) and bone mineral loss will be measured as secondary outcomes because of the reduced estrogen levels resulting from treatment with a GnRH antagonist or agonist. Furthermore, UFS-QOL will be evaluated to assess treatment effects on QOL. Evaluation of these items will clarify the usefulness and safety of using a GnRH antagonist or antagonists before LM.

This study has some limitations. First, in this study, patients and health professionals are not blinded because two study drugs use different administration routes and dosing intervals. Therefore, when interpreting study outcomes, bias will need to be considered. Second, cost-effectiveness will not be evaluated in this study. For reference, an approximate monthly cost of relugolix is higher than that of leuprorelin: relugolix costs 26,000 yen, and generic leuprorelin 1.88 mg and 3.75 mg costs 15,000 yen and 18,000 yen, respectively. Although further analysis will be needed to make a solid conclusion, the utilization of relugolix is likely a better alternative for the cost reduction.

Since the medication prescribed is approved in Japan and dispensing is part of routine clinical practice, the risks-versus-benefits of the drugs are unchanged. Therefore, we believe the results of this study will provide a safer choice and lower the burden on patients undergoing LM.

## Trial status

Recruitment began on 19 January 2022 and ended on 31 December 2023. The current protocol is version 5.0, dated on 7 July 2023.

## Data Availability

Access to anonymized patient data and statistical code will be granted to researchers at participating medical institutions. In addition, other reasonable requests will be considered.

## Abbreviations

AE: Adverse event
CI: Confidence interval
CRF: Case report form
CRO: Contract research organization
EDC: Electronic data capture
FAS: Full analysis set
FIGO: International Federation of Gynecology and Obstetrics
GnRH: Gonadotropin-releasing hormone
jRCT: Japan Registry of Clinical Trials
KKSI: Kupperman Konenki Shogai Index
LH-RH: Luteinizing hormone-releasing hormone
MRI: Magnetic resonance imaging
PPS: Per-protocol set of trial participants
QOL: Quality of life
RCT: Randomized controlled trial
UFS-QOL: Uterine Fibroid Symptom Health-Related Quality of Life
